# Methylenetetrahydrofolate reductase gene polymorphism and its association with coronary artery disease

**DOI:** 10.1590/S1516-31802007000100002

**Published:** 2007-01-04

**Authors:** Alexandre Rodrigues Guerzoni, Érika Cristina Pavarino-Bertelli, Moacir Fernandes de Godoy, Carla Renata Graça, Patrícia Matos Biselli, Dorotéia Rossi Silva Souza, Eny Maria Goloni Bertollo

**Keywords:** Coronary arteriosclerosis, Atherosclerosis, Methylenetetrahydrofolate reductase (NADPH2), Polymorphism genetic, Homocysteine, Arteriosclerose coronária, Aterosclerose, Metilenotetraidrofolato redutase (NADPH2), Polimorfismo genético, Homocisteína

## Abstract

**CONTEXT AND OBJECTIVE:**

Obstructive coronary artery disease (CAD) is characterized by the deposition of atherosclerotic plaque on the coronary artery wall. Its manifestations depend on interactions between environmental and genetic risk factors. The aim of this work was to analyze the frequency of methylenetetrahydrofolate reductase (MTHFR) C677T polymorphism in patients with CAD and its association with plasma homocysteine levels. Risk factors for CAD were also evaluated.

**DESIGN AND SETTING:**

Retrospective with blind quantitative analysis, at Hospital de Base, Faculdade de Medicina de São José do Rio Preto.

**METHODS:**

One hundred and twenty-seven individuals were studied. All completed a questionnaire to analyze risk factors for CAD. MTHFR polymorphism was investigated by restriction fragment length analysis and correlated with the number of affected arteries and degree of arterial obstruction determined by coronary cineangiography, and with plasma homocysteine levels measured by liquid chromatography/sequential mass spectrometry.

**RESULTS:**

Smoking (p = 0.02) and high-density lipoprotein cholesterol (p = 0.01) were associated with CAD. The C allele was the most prevalent in patients (0.61) and controls (0.66). There was no correlation between MTHFR/C677T polymorphism and plasma homocysteine levels. However, in patients with the TT genotype there was a correlation with the prevalence of coronary obstruction greater than 95% (p = 0.02) and the presence of two affected arteries (p = 0.04).

**CONCLUSIONS:**

The TT genotype is associated with coronary artery obstruction greater than 95% and the presence of two affected arteries. This confirms the relationship between genetic variants in specific patient subgroups and cardiovascular diseases.

## RESUMO

**CONTEXTO E OBJETIVO:**

A doença arterial coronariana (DAC) caracteriza-se pelo depósito de placa aterosclerótica na parede arterial coronária. Sua manifestação é dependente da interação entre fatores de risco ambientais e genéticos. O objetivo deste trabalho é analisar a freqüência do polimorfismo MTHFR/C677T em pacientes com doença arterial coronária e sua associação com o nível de Hcy plasmática. Fatores de risco para DAC também foram avaliados.

**TIPO DE ESTUDO:**

Retrospectivo com análise cega quantitativa, no Hospital de Base, Faculdade de Medicina de São José do Rio Preto.

**MÉTODOS:**

Foram estudados 127 indivíduos. Todos responderam a um questionário para análise dos fatores de risco para DAC. O polimorfismo MTHFR/C677T, investigado por análise de comprimento de fragmentos de restrição, foi correlacionado com número de artérias afetadas e grau de obstrução arterial, determinadas pela cinangiocoronariografia, e com o nível de Hcy plasmática.

**RESULTADOS:**

Tabagismo (p = 0,02) and HDLc (p = 0,01) foram associados com DAC. O alelo C foi o mais prevalente em pacientes (0,61) e controles (0,66; p = 0,49). O polimorfismo MTHFR/C677T não apresentou associação com níveis de Hcy plasmática. Entretanto, nos pacientes com genótipo TT observou-se a prevalência de obstrução coronariana acima de 95% (p = 0,02) e a presença de duas artérias lesadas (p = 0,04).

**CONCLUSÕES:**

Associou-se o genótipo TT com o grau de obstrução arterial coronária acima de 95% e a presença de duas artérias lesadas; confirma-se, assim, a relação de variantes genéticas em subgrupos específicos de pacientes com doenças cardiovasculares.

## INTRODUCTION

Obstructive coronary artery disease (CAD) is characterized by the deposition of atherosclerotic plaque on the coronary artery wall. This chronic disease is frequently asymptomatic, but acute events can occur as a result of instability of the atherosclerotic plaque, with consequent arterial thrombosis that results in myocardial infarction.^1^ Its manifestation depends on the interactions between environmental and genetic risk factors.^2^

Individual susceptibility to this disease has been associated with functional allelic variations. Thus, the identification of gene polymorphisms relating to the formation of atherosclerotic plaque and consequent thrombi may contribute towards developing early diagnostic methods and guiding preventive procedures.^2-4^

The gene that codes for the methylenetetrahydrofolate reductase enzyme (MTHFR), which is involved in metabolizing homocysteine (Hcy), is of great interest in clinical practice.^2,5,6^ Hcy, an amino acid derived from protein catabolism, is present in the plasma in several forms, with the greatest proportion (70%) bound to albumin.^3^ High levels of Hcy (hyperhomocysteinemia) have been identified as a risk factor for atherosclerosis.^2,3,7-9^ The serum concentration of Hcy is high in 40% of patients suffering from coronary cerebral or peripheral artery diseases, while in control groups only 15% of individuals present with elevated levels.^9^

The mechanism for the vascular lesions induced by hyperhomocysteinemia remains unclear. Experimental evidence suggests that Hcy may be involved in atherogenesis and thrombogenesis, leading to hyperplasia of cell tissue and fibrosis. Additionally, it facilitates the vascular oxidative process, alters the co agulation system and reduces the vasomotor regulation of the endothelium.^6,7,10,11^

A moderate increase in Hcy is more generally associated with the cytosine-to-thymine substitution at nucleotide 677 (C677T) of the MTHFR gene, thereby leading to an alanine-for-valine substitution. This alteration is associated with increased enzyme thermolability, causing a 50% reduction in its normal activity.^7,11^

## OBJECTIVE

This study had the aim of analyzing the frequency of C677T polymorphism in the MTHFR gene in patients with obstructive coronary artery lesions, in comparison with individuals without angiographic evidence of the disease. In addition, it evaluated the association between this polymorphism and the number of affected arteries and degree of arterial obstruction and plasma Hcy levels. Risk factors for CAD were also evaluated.

## METHODS

For this retrospective study, after obtaining informed consent, we recruited 127 Caucasian individuals (83 men and 44 women; mean age = 60 ± 12 years). Although in Brazil there is widespread miscegenation, we considered as Caucasians those individuals who had no other ethnic group in the three generations preceding them.^4^ All of them underwent coronary cineangiography because of clinical indications relating to angina or positive induced ischemia tests. Cineangiography identified 91 individuals with CAD (65 men and 26 women; mean age 60.3 ± 12 years) and 36 control subjects, without signs of the disease (22 men and 14 women; mean age 60.4 ± 12 years). The disease was defined and identified in patients according to current criteria^1^. The coronary cineangiography results were analyzed by two independent observers in a quantitative blind analysis in the Hemodynamics and Interventional Cardiology Section of Faculdade de Medicina de São José do Rio Preto (Famerp), São José do Rio Preto, São Paulo, Brazil. Individuals who had undergone heart surgery and those with coronary prosthesis were excluded from the study.

All participants completed a questionnaire to analyze risk factors such as diabetes (patients who were using antidiabetic medication or presented with blood sugar levels greater than 126 mg/dl), hypertension (patients who were using specific medications or had blood pressures higher than 140/90 mmHg), sedentary patients (patients who did not do regular, controlled physical exercise), alcoho lism (patients who were consuming alcohol at a defined frequency, without ta king into account the quantity) and smoking (minimum of five cigarettes per day). Blood samples were obtained after 12 hours of fasting, to analyze the lipid profile. The analysis included the serum levels of total cholesterol (TC) and the fractions of low-density lipoprotein cholesterol (LDLc), high-density lipoprotein cholesterol (HDLc), very low-density lipoprotein cholesterol (VLDLc) and triglycerides (TG), measured using the enzymatic colorimetric assay method and Friedewald's formula to calculate LDLc. Measurement of Hcy was performed by liquid chromatography/sequential mass spectrometry. Genomic DNA was obtained form leukocytes,^12^ followed by its amplification by means of the polymerase chain reaction (PCR) using the primers described by Bova et al.^9^ and restriction enzyme digestion with *Hinf*I. The digested product was submitted to electrophoresis on 9.6% polyacrylamide gel and the digestion products were viewed by staining with silver nitrate.

The data obtained were statistically assessed using the chi-squared, Fisher's exact, Kruskal-Wallis or Mann-Whitney tests, depending on the type of variable analyzed, by means of the Graphpad computer software, and using logistic regression. An alpha error of 5% was considered acceptable, and therefore p-values less than or equal to 0.05 were considered significant.^13^

In accordance with the guidelines on human research from the Brazilian Health Ministry, approval was obtained for this study from the Research Ethics Committee of Famerp, São José do Rio Preto, State of São Paulo, and from the National Research Commission in Brasilia, Federal District.

## RESULTS

Table 1 shows characteristics of patients and controls and their lipid profile. Prior diseases identified in the CAD group (91 patients) included high blood pressure in 81% of the individuals and diabetes in 25%. In the control subjects the rates were 60% and 8%, respectively. Seventy-one percent of the patients reported they were smokers; hence there was no significant difference in comparison with the controls (55%; p = 0.13). Serum levels for the lipid profile remained within the reference range in both groups except for HDLc, for which levels of less than 40 mg/dl were significantly more frequent in the patients (53%) than in the control subjects (30%, p = 0.03).

**Table 1.** Characterization of patients with coronary artery disease and controls who underwent angiography, and the respective statistical significance between the two groups (p-value)

Profile	Patients (n = 91) n (%)	Controls (n = 36) n (%)	p-value
**Hypertension**	74 (81)	24 (60)	0.124
**Diabetes**	23 (25)	3 (8)	0.059
**Sedentary lifestyle**	36 (39)	13 (36)	0.875
**Smoker**	65 (71)	20 (55)	0.133
**Angina**	83 (91)	31 (86)	0.597
**Ventricular involvement**	33 (36)	10 (27)	0.482
**Alcoholism**	32 (35)	11 (30)	0.774
**TC (> 200 mg/dl)**	36 (39)	11 (30)	0.457
**LDLc (> 130 mg/dl)**	31 (34)	11 (30)	0.865
**HDLc (< 40 mg/dl)**	49 (53)	11 (30)	0.03*
**VLDLc (> 30 mg/dl)**	30 (33)	14 (38)	0.671
**TG (> 150 mg/dl)**	33 (36)	15 (41)	0.717

*TC = total cholesterol; LDLc = low-density lipoprotein cholesterol fraction; HDLc = high-density lipoprotein cholesterol fraction; VLDLc = very low-density lipoprotein cholesterol fraction; TG = triglycerides.*

* *p < 0.05.*

Logistic regression indicated associations between CAD and smoking (p = 0.02) and HDLc (p = 0.01). No associations were seen between CAD and diabetes (p = 0.1359), hypertension (p = 0.1065) or C677T polymorphism (p = 0.56), in the individuals studied.

The mean values relating to the lipoprotein profile showed slightly higher levels of TC and LDLc in patients (193 ± 61 mg/dl and 124 ± 50 mg/dl respectively) than in controls (187 ± 39 mg/dl and 119 ± 31 mg/dl). Even so, both groups presented with levels within the reference ranges. On the other hand, the lower mean values for HDLc were similar in the two groups (38 ± 11 mg/dl and 38 ± 7 mg/dl, respectively).

Analysis of the allelic and genotypic frequencies of C677T polymorphism (Table 2) demonstrated that the most prevalent allele type was the C allele, in both patients (0.61) and controls (0.66). The most frequent genotype was the CT genotype, in both groups (65% and 55%, respectively), followed by the CC genotype (28% and 39%, respectively). There was no significant difference between the groups with regard to the allelic and genotypic frequencies.

**Table 2.** Distribution of the allelic and genotypic frequencies of C677T polymorphism in 127 individuals who underwent angiography

	Patients (n = 91)		Controls (n= 36)		p-value
**Genotype**	**n**	**%**	**n**	**%**	
CC	26	28	14	39	0.360
CT	59	65	20	55	0.442
TT	6	7	2	6	0.851
T allele	71	0.39*	24	0.34*	0.485

* *absolute frequency.*

A degree of arterial obstruction greater than 95% and presence of two damaged arte ries seemed to be associated with the TT genotype, in comparison with the other genotypes, u sing Fisher's exact test (p = 0.029, Table 3; and p = 0.04, Table 4). Twenty-four patients (26%) presented obstruction in one artery, 38 (42%) in two arteries and 29 (32%) in three arteries. The artery affected was the anterior descen ding artery in 84 cases (92%), followed by the right coronary artery in 71 cases (78%) and the circumflex artery in 50 cases (55%).

**Table 3.** Coronary artery involvement in patients, in relation to C677T polymorphism, and the respective statistical significance (p-value)

Genotype	< 50%	50-75%	75-95%	> 95%
	n (p-value)	n (p-value)	n (p-value)	n (p-value)
CC	2 (0.861)	4 (0.596)	14 (0.269)	6 (0.908)
CT	6 (0.708)	14 (0.523)	27 (0.906)	12 (0.366)
TT	0 (0.967)	1 (0.681)	1 (0.212)	4 (0.029)*

* *p < 0.05.*

**Table 4.** Distribution of the genotypic frequencies of methylenetetrahydrofolate reductase/ C677T (MTHFR/C677T) polymorphism, in relation to the number of arteries affected

Genotype	Number of affected arteries		
	1 n = 24	2 n = 38	3 n = 29
	n (p-value)	n (p-value)	n (p-value)
CC	7 (0.48)	9 (0.28)	10 (0.25)
CT	16 (0.41)	24 (0.43)	19 (0.46)
TT	1 (0.46)	5 (0.04)*	0 (0.10)

* *p < 0.05.*

Analysis of mean Hcy concentrations demonstrated higher levels in patients (20.53 μmol/l) than in controls (16.29 μmol/l) but the difference was not statistically significant (p = 0.25); in relation to C677T polymorphism the mean Hcy levels were higher in patients with the CT genotype (23.95 μmol/l) and TT genotype (15.63 μmol/l) than in the controls (16.39 μmol/l and 3.15 μmol/l, respectively) but again without statistical significance (Table 5). In addition, no associations were observed between the presence of the T allele and the number of patients with raised Hcy levels in either the patient or control group.

**Table 5.** Analysis of homocysteine (Hcy) levels between patients and controls, in relation to C677T polymorphism, and the respective statistical significance (p-value)

	Patients Hcy μmol/l		Controls Hcy μmol/l		p-value
	**Mean**	**Standard deviation**	**Mean**	**Standard deviation**	
	20.53	19.17	16.29	8.92	0.25
**Genotype**					
CC	16.96	10.43	16.99	7.95	0.99
CT	23.95	24.41	16.39	9.56	0.12
TT	15.63	6.24	3.15	_*_	_*_

* *Analysis not performed, because the control group had only one individual with the TT genotype.*

## DISCUSSION

In this study, MTHFR/C677T polymorphism did not differentiate between patients and controls. Moreover, CAD was mainly observed in patients with decreased HDLc levels and those who were smokers (logistic regression), which is in agreement with other studies.^14,15^ On the other hand, the similar levels of TC, LDLc, VLDLc and TG in the two groups found in the present study are discordant with other published studies.^16,17^

The other risk factors and clinical signs, including high blood pressure, lack of exercise, ventricular involvement and alcoholism were also similar between the groups. It is possible that the selection of controls (with negative angiography for coronary artery obstruction) in the cardiology outpatient clinic may have contributed towards the very high prevalences of hypertension (60%) and angina (86%).^18^

C677T polymorphism of the MTHFR gene has been designated a strong candidate for increased risk of vascular disease because of its influence on Hcy levels, which are an independent risk factor for atherosclerosis,^2,7,9-11,16,19^ such that the T allele is significantly more frequent in patients with CAD (0.42) than in controls (0.33; p = 0.0001).^20^ However, individual and meta-analytical studies on approximately 6000 patients have not confirmed this hypothesis.^7,21,22^ Moreover, although the present study showed similar prevalences for the T allele in patients (0.39) and controls (0.34), this finding corroborates with the literature.^23^ The TT genotype, with a prevalence of 7% in patients, was associated with a degree of obstruction greater than 95%, which is in agreement with a study on CAD patients by Morita et al.,^20^ in 1997. There has also been reference to associations between the T allele and peripheral vascular disease, but with decreased frequencies (0.31 in patients and 0.27 in controls).^16^ Nevertheless, patients with atherosclerosis of the carotid artery have presented with significantly higher frequencies of the T allele (0.47) than in control subjects (0.27; p < 0.02).^9^

Studies have shown that there is a relationship between the C677T genetic variant,^21,23^ Hcy level and myocardial infarction. Higher plasma Hcy concentrations have particularly been detected in patients with CAD and the TT genotype.^23^

Some studies have been conducted on Brazilian patients at risk of vascular thrombosis. High Hcy levels were detected in patients with the MTHFR 677TT genotype; however, this was not associated with increased risk of the disease.^4,24,25^

Concentrations greater than or equal to 15.6 μgmol/l have been associated with greater risk of myocardial infarction than in individuals with levels less than 10.0 μgmol/l (odds ratio = 2.3; 95% confidence interval range from 0.94 to 5.64).^23^

Studies have also revealed a relationship between the TT genotype and decreased Hcy levels in patients with daily folate supplementation of approximately 400 μg together with vitamins B_12_ and B_6._^8^ Schwartz et al.^23^ investigated the relationships between the risk of myocardial infarction and serum folate levels, vitamin B_12_, Hcy and C677T polymorphism. In that study, women with folate levels greater than or equal to 8.39 nmol/l presented with decreased prevalence of myocardial infarction in comparison with women whose folate levels were less than 5.27 nmol/l (odds ratio = 0.54; 95% confidence interval range from 0.23 to 1.28). However, there were no significant differences with regard to vitamin B_12_ levels (odds ratio = 0.90, 95% confidence interval range from 0.31 to 2.29). Thus, even though folic acid, vitamin B_6_ and vitamin B_12_ supplementation can reduce Hcy levels,^26^ such intake is neglected by the general population and by healthcare professionals.

Although the present study did not demonstrate any associations between Hcy, CAD and C677T polymorphism, the higher mean Hcy levels in patients with the T allele suggest that significant results might be found in larger cohorts. Thus, further studies are needed, using larger series to analyze the real risk exerted by the presence of C677T polymorphism and its relationship with risk factors for CAD.

One difficulty in this study was the ethnic characterization. A great number of individuals were excluded because they were unable to unequivocally affirm their ethnic origin. Another difficulty was the cost relating to Hcy measurements, which was resolved through collaboration with the Thonson Laboratory of the Chemistry Department of the University of Campinas.

Because of the importance of studies on different populations and with greater sample sizes, the authors intend to continue investigating the relationship between CAD and the MTHFR gene, as well as other genes related to folate metabolism (methionine reductase and methionine synthetase). Furthermore, the importance of B_6_ and B_12_ vitamin intake and folic acid intake will be studied in an attempt to analyze the influence of these variables.

## CONCLUSIONS

The present study identified associations between the TT genotype and coronary artery obstruction of greater than 95% and presence of two injured arteries. Additionally, the relationship between genetic variants in specific patient subgroups and cardiovascular diseases was confirmed. However, the most important contribution of the present study was the finding of an association between C677T polymorphism of the MFHFR gene and the severity of the disease.
